# Harnessing the capabilities of an electronic health record system to enhance surveillance for Middle East respiratory syndrome in an acute hospital

**DOI:** 10.1017/ash.2025.83

**Published:** 2025-09-03

**Authors:** Aung Hein Aung, Win Mar Kyaw, Ong Lay See, Joey Phua Zhi Jing, Sim Jian Wei, Tang Ying Wei, Lim Wei-Yen, Chow Li Ping Angela

## Abstract

**Objectives:** The recent experience of the COVID-19 pandemic emphasized the critical need for a surveillance system to alert healthcare facilities about the admission of patients with emerging infectious diseases (EID), thereby preventing nosocomial transmissions. **Methods:** Tan Tock Seng Hospital, an 1800-bed acute tertiary-care hospital in Singapore, transitioned to a new- generation electronic medical record system, Epic, in August 2022. Leveraging the system’s capabilities, we developed an algorithm to generate the line-list of suspected Middle East Respiratory Syndrome (MERS) patients, in alignment with the screening guidelines provided by Singapore’s Ministry of Health. The algorithm first identifies patients who presented within 14 days (maximum incubation period) of their travel to Arabic peninsular countries. This information is documented by the emergency department’s triage nurses. Additionally, patients with suspected MERS indicated in the problem list or diagnosis by attending clinicians, particularly emergency-medicine physicians or infectious-disease physicians, are included. Furthermore, patients who are ordered for a MERS- Coronavirus polymerase chain reaction test, are identified. The algorithm can also be further modified as and when the case definition of the EID changes. **Results:** The surveillance report constructed with Epic algorithm can be scheduled for daily generation or generated on demand within a few minutes. This newer approach is more time- and resource-efficient compared to the manual surveillance process, which necessitates at least three staff members to engage in a series of prolonged manual processes. The report, by extracting information directly from Epic in near real-time, also minimizes the likelihood of errors that may occur during the manual process. Subsequently, the team of epidemiologists identifies the suspected MERS patients form the generated report and efficiently follow up them until a diagnosis of MERS is excluded. **Conclusions:** Harnessing Epic’s capabilities, we constructed an algorithm to efficiently and swiftly identify suspected MERS patients, enabling the timely implementation of infection prevention strategies to prevent nosocomial transmission.

